# Prevalence of mental disorders, psychosocial distress, and perceived need for psychosocial support in cancer patients and their relatives stratified by biopsychosocial factors: rationale, study design, and methods of a prospective multi-center observational cohort study (LUPE study)

**DOI:** 10.3389/fpsyg.2023.1125545

**Published:** 2023-04-20

**Authors:** Anja Mehnert-Theuerkauf, Julia Marie Hufeld, Peter Esser, Ute Goerling, Myriel Hermann, Tanja Zimmermann, Hannah Reuter, Jochen Ernst

**Affiliations:** ^1^Department of Medical Psychology and Medical Sociology, University Medical Center Leipzig, Leipzig, Germany; ^2^Charité – Universitätsmedizin Berlin, Corporate Member of Freie Universität Berlin, Humboldt-Universität zu Berlin, Berlin Institute of Health, Charité Comprehensive Cancer Center, Berlin, Germany; ^3^Department of Psychosomatic Medicine and Psychotherapy, Hannover Medical School, Hannover, Germany

**Keywords:** psychosocial distress, cancer patient, relatives, psychosocial support, psycho-oncology, socioeconomic status, survivorship, mental disorders

## Abstract

**Background:**

Despite remarkable progress, cancer remains a life-threatening disease for millions of people worldwide, also resulting in significant psychosocial limitations. High-quality, comprehensive cancer care requires patient and family involvement and the provision of needs-based, targeted psychosocial services. Although progress has been made in understanding the occurrence of mental comorbidity and psychosocial distress in cancer patients, comparatively little is known about the course of psychological comorbidity and psychosocial distress in early survivorship among patients and their families. We therefore aim to estimate the prevalence of mental disorders according to the DSM-5, psychosocial distress, perceived needs for psychosocial support and utilization of psychosocial support offers in newly diagnosed cancer patients and their relatives, taking into account potential contributing biopsychosocial factors for the occurrence of psychological comorbidity.

**Methods/design:**

This study follows a prospective multi-center observational cohort design across four measurement time points: within 2 months after cancer diagnosis (t1), and in the follow-up period at 6 months (t2), at 12 months (t3), and at 18 months (t4) after t1. Patients older than 18 years who have a confirmed initial diagnosis of a malignant solid tumor and are scheduled for cancer treatment at one of the participating cancer centers are eligible for study participation. Relatives of eligible patients are also eligible for study participation if they are older than 18 years. Patients are interviewed using the Structured Clinical Interview for DSM-5 Disorders (SCID-5-CV). In addition, patients and relatives receive a set of validated questionnaires at each measurement time point, covering comorbid conditions and functional performance, perceived psychological distress and quality of life, partnership aspects and social relationships, supportive care needs and use of psychosocial support services, health literacy, and health behavior and meaning in life.

**Discussion:**

This prospective multi-center observational cohort study has a major focus on increasing quality of care and quality of life in cancer survivors through providing rigorous longitudinal data for the development and implementation of target group-specific psychosocial support services.

**Trial registration:**

NCT04620564, date of registration 9/11/2020; DKG OnkoZert: Registrier-No.: ST-U134, date of registration 5/11/2021.

## Introduction

Despite remarkable progress in cancer research, diagnostics and treatment, cancer continues to affect millions of people worldwide as a life-threatening disease, associated with invasive interventions and often irreversible impairments (Sung et al., 2021). The increasing incidence of cancer in many countries will lead to a higher prevalence of cancer survivors in the coming years. Survivors in different age generations are affected to varying degrees by pre-existing as well as disease- and treatment-related physical and psychological comorbidities that are significant in their frequency and severity (Singer et al., 2013; Hartung et al., 2017; Götze et al., 2018, 2019; Smitherman et al., 2018; Niedzwiedz et al., 2019; Renzi et al., 2019; Chao et al., 2020). Mental comorbidity is understood as the presence of a mental disorder in addition to cancer that meets the diagnostic criteria of the Diagnostic and Statistical Manual of Mental Disorders (DSM) (American Psychiatric Association [APA], 2013) or the International Classification of Diseases (ICD) (World Health Organization [WHO], 2022). The meta-analysis by Mitchell et al. (2011) found pooled prevalence rates of 16.3% for depression, 19.4% for adjustment disorders, and 10.3% for anxiety disorders in over 70 international studies and more than 10,000 cancer patients in oncology and haemato-oncology settings. Combined diagnoses were common; the prevalence for any mood and/or anxiety disorder was 38.2%. Our research group found a 4-week prevalence of 31.8% for any mental disorder in a large epidemiological study in Germany, with anxiety disorders (11.5%) and adjustment disorders (11.1%) being most prevalent (Mehnert et al., 2014). The 12-month prevalence of any mental disorder here was 39.4% and the lifetime prevalence was 56.3% (Kuhnt et al., 2016). Over a 12-month period anxiety disorders and mood disorders were the most frequent mental disorders.

In distinction to the presence of a mental disorder, a large proportion of cancer patients suffer from a high degree of perceived distress, including anxiety and depressive mood (Herschbach et al., 2020; Bach et al., 2022). In a representative sample across all major tumor entities, 52% of patients reported high psychological distress. The highest distress levels were found in patients with female genital cancers or pancreatic cancer (Mehnert et al., 2018). In a sample of 10,153 patients with various types of cancer, Linden et al. (2012) found that 19% of patients suffered from clinical anxiety and further 23% from subclinical anxiety symptoms. In addition, 13% of the patients had clinical symptoms of depression and another 16.5% had subclinical symptoms.

Psychosocial adjustment to cancer varies widely, with some patients improving over time and others deteriorating. Studies on the course of distress, anxiety, and depression symptoms show different trajectories depending on individual coping strategies, long-term and late effects of cancer treatment, and psychosocial stressors before and during the survivorship phase (Lam et al., 2013; Hellstadius et al., 2017; Dionisi-Vici et al., 2021). Lopes et al. (2022) studied the course of anxiety and depressive symptoms in a cohort of 506 breast cancer patients who were followed for 5 years after cancer diagnosis. The authors identified three trajectories of anxiety symptoms and three of depressive symptoms. The trajectories of high anxiety and high depression during the 5 years of follow-up were equally frequent. One in five women was affected by each trajectory. The other two anxiety and two depression trajectories showed a decline in scores over time: one trajectory with mid-range scores and one with low scores for anxiety symptoms, and similarly, one mid-range scores and one low scores trajectory, for depression symptoms.

Higher levels of psychosocial distress have been found to be associated with significantly lower quality of life, higher morbidity and mortality (Russ et al., 2012; Walker et al., 2020, 2021; Varela-Moreno et al., 2022) and higher costs to the health system (Chiu et al., 2017). Correlates of psychological distress and mental comorbidity in cancer populations include female gender, poor physical functioning that is often associated with advanced disease status and more invasive cancer treatments, less social support and socioeconomic status (SES), measured by education, income, and/or occupation (Singer et al., 2013; Syrowatka et al., 2017; Sauer et al., 2019).

In particular, the relationship between SES and health, especially mental health, has been extensively demonstrated for other diseases (Fryers et al., 2003; Mirza and Jenkins, 2004; Hanel et al., 2009; Moreno-Peral et al., 2014; Mackenbach, 2019; Marmot, 2019; Toussaint and Herzog, 2020; Barbek et al., 2022). Research suggests a higher prevalence of psychological comorbidities and health burden in patients with lower SES, but data for cancer populations are insufficient. There are multiple reasons why social inequalities in health lead to different outcomes in terms of morbidity, mortality and quality of life. It is likely that patients with low SES are more likely to be exposed to negative psychosocial factors (Siegrist and Marmot, 2004). Behavioral and lifestyle factors play an important role in relation to tobacco and alcohol consumption, diet and physical activity. Furthermore, SES has an influence on health literacy, access to health information and the use of health services (Mackenbach et al., 2008). Structural aspects of the health system also affect SES-specific utilization behavior, such as access barriers to health services for people with lower SES.

Although progress has been made in understanding the occurrence of mental comorbidity and psychosocial distress in cancer patients, comparatively little is known about the course of psychological comorbidity and psychosocial distress in the early survivorship phase in patients and their relatives. Relatives and informal caregivers experience high levels of emotional distress such as anxiety, helplessness and hopelessness, depressed mood, and impaired quality of life (Sklenarova et al., 2015; Thana et al., 2021; Goodwin et al., 2022). Acute, long-term and late physical and psychosocial impairments due to the partner’s cancer, role changes, difficulties in coping with the disease or impaired family interaction processes are stressors for the relatives (Hagedoorn et al., 2008). Many informal caregivers also face a difficult task: while they are often the main source of emotional and practical support for the sick person and are involved in medical decision-making (Ernst et al., 2017; Laidsaar-Powell et al., 2017; Weissflog et al., 2017; Cincidda et al., 2022), they themselves have a high psychosocial burden that requires support (Zimmermann, 2022).

Hu et al. (2023) showed a higher risk of any mental disorder in spouses of cancer patients compared to spouses of individuals without cancer in a binational study in Denmark and Sweden. The risk of first-onset mental disorders increased by 30% during the first year after cancer diagnosis, especially for depression and stress-related disorders. The risk increase was thus particularly high during the first year following cancer diagnosis and persisted during the entire follow-up.

Therefore, comprehensive cancer carer requires the involvement of the patient’s family and social environment and the provision of needs-based psychosocial services for relatives. Considering biopsychosocial factors such as SES as influencing factors on distress and mental comorbidity according to the new diagnostic criteria (DSM-5) will provide much needed data for more targeted psychosocial care for groups with specific needs. The data will most likely also reveal factors that are important for strengthening resilience and resource orientation.

### Objectives and scientific aims

Our primary study aims are to (i) estimate the prevalence of mental disorders according to the DSM-5 (APA) in newly diagnosed cancer patients a few weeks after cancer diagnosis and over a follow-up period of 18 months; and (ii) to assess the frequency and extent of psychosocial distress, perceived needs for psychosocial support and utilization of psychosocial support offers in newly diagnosed cancer patients and their relatives a few weeks after cancer diagnosis and over a follow-up period of 18 months.

In particular, we take into account demographic factors such as age, sex, SES and cancer-related factors as potential contributing factors for the occurrence of psychosocial distress and supportive care needs in both patients and relatives. We further aim to identify and analyze moderating and mediating as well as associated factors such as dyadic coping, quality of life, fear of recurrence, adherence, and satisfaction with medical and psychosocial care for the occurrence of mental disorders, psychological distress, and supportive care needs.

## Materials and methods

### Study design

The study follows a prospective multi-center observational cohort design across four measurement time points: within 2 months after cancer diagnosis (t1), and in the follow-up period at 6 months (t2), at 12 months (t3), and at 18 months (t4) after t1. The study is being conducted at the university cancer centers of the three main German study centers in Leipzig, Berlin and Hannover as well as at cooperating cancer centers in Braunschweig, Dresden, and Göttingen. The study design follows both STROBE statement for observational studies (Vandenbroucke et al., 2007) and Patient-Centered Outcomes Research Institute (PCORI) (Selby et al., 2012) methodology standards, including patient engagement, research questions and methods, data integrity and analysis, preventing and handling of missing data and dissemination of and implementation of study results.

The study is funded by the German Cancer Aid Foundation (Stiftung Deutsche Krebshilfe) (funding numbers: 70113544, 70113611, and 70113613) and additionally financially supported by the Department of Medical Psychology and Medical Sociology at the University Medical Center of Leipzig, by the Charité Comprehensive Cancer Center, Berlin, and the Department of Psychosomatic Medicine and Psychotherapy, Hannover Medical School, Hannover.

### Study participants, setting, and recruitment

Patients older than 18 years who have a confirmed initial diagnosis of a malignant solid tumor according to the medical record and are scheduled for cancer treatment at one of the participating cancer centers are eligible for study participation. Further inclusion criteria are: sufficient knowledge of the German language, physical, psychological, and cognitive ability for study participation, and written informed consent for study participation.

Relatives of eligible patients are also eligible for study participation if they are older than 18 years and provide written informed consent for study participation.

Study enrolment began in April 2020 and ended in July 2022 (t1). Follow-up assessments (t2–t4) are projected to end in January 2024. Patients are consecutively screened by the study staff for inclusion criteria at the participating cancer centers. Eligible patients are contacted personally by trained study staff and receive written educational material describing the study. If it is not possible for the study team to contact the patient at the cancer center, for example due to COVID-19 patient safety restrictions, patients receive a letter sent at their home address. If patients have given their written informed consent to be contacted, they receive all documents and a phone call, in which they are also asked to name a relative. The relative should be from the patient’s immediate living environment, if possible. If the patient has consented, the study team receives the contact details of the relative to invite him/her to participate in the study as well.

### Data collection

Patients fulfilling the inclusion criteria who agreed to participate were interviewed using the German adaptation of the Structured Clinical Interview for DSM-5 Disorders (SCID-5) (First et al., 2015; Beesdo-Baum et al., 2019). The SCID-5 was administered face-to-face by trained study staff during inpatient care or by telephone due to COVID-19 patient safety restrictions (t1). At follow-up (t2–t4), the SCID-5 was performed usually by telephone.

In addition to the SCID-5, participating patients receive a set of validated questionnaires and a stamped return envelope. They are asked to complete the study questionnaires within 10 days and return it to the local study center. Alternatively, patients will be offered to complete the study questionnaires online via an individualized link using the LimeSurvey software (LimeSurvey GmbH, 2015). For the follow-up measurements (t2–t4), patients again receive the study questionnaires with a stamped return envelope by post or an individualized link to LimeSurvey.

Relatives are not interviewed with the SCID-5. They receive a set of validated study questionnaires according to the same data collection scheme at all measurement times (t1–t4) either by post with a prepaid envelope or via an individualized link to LimeSurvey.

Both patients and relatives will systematically receive a telephone reminder from the study team after 14 days and after 3 weeks if they have not completed and returned the questionnaire by then. Patients and relatives are also offered support by the trained study team if they have difficulties completing the study questionnaires.

### Study measures

Table 1 gives an overview over the study measures including the SCID-5 clinical interview and self-report questionnaires that will be completed by patients and relatives.

**TABLE 1:** Study assessment schedule and study measures.

		Assessment schedule and sample			
**Study measures**		**t1** **<8 weeks post primary cancer disclosure**	**t2** **6 months post t1**	**t3** **12 months post t1**	**t4** **18 months post t1**
		**Patients (P), relatives (R)**	**Patients (P), relatives (R)**	**Patients (P), relatives (R)**	**Patients (P), relatives (R)**
**Demographics, medical history, comorbid conditions, and functional performance**					
SES	Socioeconomic status (screening)	P, R	–	–	–
	Demographic characteristics	P, R	P, R (update)	P, R (update)	P, R (update)
	Medical characteristics, medical records	P	P (update)	P (update)	P (update)
CCC-18	Chronic comorbid conditions	P	–	–	–
	Corona related stress – single Item	P, R	P, R	P, R	P, R
KPS	Karnofsky performance status scale	P	P	P	P
	Mental disorders	P	P	P	P
SCID-5-CV	Structured clinical interview for DSM-5 disorders – clinical version	P	P	P	P
**Psychological distress and quality of life**					
DT	Distress thermometer	P, R	P, R	P, R	P, R
DT-PL	Distress problem list	P, R	–	–	–
ET	Emotion thermometers	P, R	P, R	P, R	P, R
PHQ-9	Patient health questionnaire depression module	P, R	P, R	P, R	P, R
GAD-7	Generalized anxiety disorder screener	P, R	P, R	P, R	P, R
FoP-Q-SF	Fear of progression questionnaire – short version	P, R	P, R	P, R	P, R
EORTC-QLQ-C30	European organization for research and treatment of cancer quality of life questionnaire	P	P	P	P
SF-8	Short-form health survey	P, R	P, R	P, R	P, R
**Partnership aspects and social relationships**					
ISSS-8	Illness-specific social support scale – short version	P, R	P, R	P, R	P, R
DCI	Dyadic coping inventory	P, R	P, R	P, R	P, R
QMI	Quality of marriage index	P, R	P, R	P, R	P, R
LS-3	Loneliness scale	P, R	P, R	P, R	P, R
NSSS-SD	New sexual satisfaction scale – short version			P	
SQoL	Sexual quality of life			P	
**Supportive care needs and use of psychosocial support services**					
SCNS-items	Supportive care needs survey – single items	P, R	P, R	P, R	P, R
	Psychosocial care needs	P, R	P, R	P, R	P, R
	Opportunity to talk to someone	P, R	P, R	P, R	P, R
	Utilization of psychosocial support services	P, R	P, R	P, R	P, R
	Attitude toward psychosocial support	P, R	P, R	P, R	P, R
	Participation in an oncological rehabilitation program		P	P	P
SCCC	Satisfaction with comprehensive cancer care	P	P		P
**Health literacy, health behavior, and perceived stigma**					
HLS-EU-Q16	Health literacy questionnaire	P			P
AAQ	Adherence assessment questionnaire		P, R	P, R	
	Questions for the assessment of health behavior		P	P	P
SIS-D	Stigma impact scale	P, R		P, R	
**Meaning and purpose in life**					
SMiLE	Schedule for meaning in life evaluation	P, R		P, R	
LAP-R-items	Life attitude profile-revised – single items	P, R		P, R	

### Sociodemographic and medical characteristics

Sociodemographic data such as age, sex, nationality, religion, marital status and partnership, children, school education, vocational training, monthly household income, employment status, occupational situation, housing situation, and residential area were collected during the SCID-5 telephone interview with the patients. The same demographic characteristics and additionally the relationship to the patient were collected from the relatives using a standardized questionnaire.

Socioeconomic status is determined by indicators including education, income, and/or occupation according to Winkler and Stolzenberg (2000).

Medical information on tumor entity, date of initial diagnosis and time since initial diagnosis, Union for International Cancer Control (UICC) disease stage, information on remission, recurrence and progression of cancer, metastases, and current cancer treatments is collected from medical records and standardized questionnaires.

### Comorbid conditions and functional performance

#### Comorbidity of chronic diseases

We assess the number and severity of chronic diseases using a modified version of a self-report instrument developed by Bayliss et al. (2005). The original questionnaire includes a list of 23 common chronic conditions. Validation against the standard criterion of medical record review showed a mean sensitivity of 75% and a mean specificity of 92%. For our study, the specificity of the original scale was reduced by grouping similar disorders together. Two additional comorbidities were added: mental disorders and sensory disturbances. The modified questionnaire contained a final 18 conditions: hypertension, asthma, lung disease, diabetes, thyroid disease, chronic back pain, rheumatism, osteoarthritis, osteoporosis, colon problems, stomach problems, kidney disease, sensory disturbances, heart disease, stroke, neurological disease, eye disorders, and mental disorders. For each condition, respondents indicate whether they currently have the condition and whether the condition affects their daily activities from 1 (“not at all”) to 5 (“very much”). A comorbidity index can be calculated, which represents the degree of morbidity. The total score ranges from 0 to 90 and represents the sum of the conditions weighted by the degree of impairment assigned to each condition (Bayliss et al., 2009).

#### Karnofsky Performance Status Scale

We use the Karnofsky Performance Status Scale (KPS) (Karnofsky and Burchenal, 1949) to rate the ability of a patient to perform usual activities. A person is evaluated on a score ranging from 0 to 100, where 0 is “dead” and 100 is “normal, no complaints, and no signs of disease.” The lower the Karnofsky score, the worse the survival for most severe diseases such as cancer.

### Mental disorders

#### Structured Clinical Interview for DSM-5 Disorders – clinical version

The Structured Clinical Interview for DSM-5 Disorders – Clinical Version (SCID-5-CV) (First et al., 2015) is a semi-structured interview for diagnosing mental disorders according to the DSM-5. Each DSM-5 criterion is assigned corresponding interview questions to assist the interviewer in assessing the criterion. The interview covers the most common DSM-5 diagnoses encountered in the clinical setting. In this study, 7 of the 10 modules were asked: (i) mood episodes and persistent depressive disorder, (ii) differential diagnosis of mood disorders, (iii) substance use disorders, (iv) anxiety disorders, (v) obsessive-compulsive and related disorders and posttraumatic stress disorder (PTSD), (vi) screening questions for other disorders, and (vii) adjustment disorders.

### Psychological distress and quality of life

#### Distress thermometer and problem list

We measure psychological distress by using the validated German version of the Distress Thermometer (DT) (Roth et al., 1998; Mehnert et al., 2006b). The DT is a valid, reliable, and widely used screening measure. The screening contains a single−item visual analog scale ranging from 0 (“no distress”) to 10 (“extreme distress”) to quantify the global level of distress experienced in the past week including the current day and a standardized problem checklist containing 36 potential causes of distress (yes/no questions) that are grouped into 5 categories including physical problems (21 items), practical (5), family (2), emotional problems (6), and spiritual/religious concerns (2). A score of ≥5 at the visual analog scale is recommended as a cutoff for a clinically significant level of distress (Mehnert et al., 2006b; Donovan et al., 2014).

#### Emotion thermometers

We measure emotional responses beyond core distress during the past week, including the current day using the validated German version of the Emotion Thermometers (ET) as a multi-domain screening (Mitchell et al., 2010a,b; Hinz et al., 2019). In addition to the general distress screening, the ET consist of four visual analog scales that measure anxiety (AnxT), depression (DepT), anger (AngT), and the need for help (HelpT). Similar to the DT, all dimensions are to be rated on the visual analog scale with the anchors 0 (“none”) and 10 (“extreme”). Higher scores indicate higher emotional distress or need for help.

We additionally assess the current burden of the COVID-19 pandemic using a 5-point Likert scale ranging from 1 (“not distressed at all”) to 5 (“highly distressed”).

#### Patient Health Questionnaire Depression Module

We use the validated German version of the Patient Health Questionnaire Depression Module (PHQ-9) (Kroenke et al., 2001; Löwe et al., 2004), the 9-item depression module from the Patient Health Questionnaire (PHQ). It was designed to score each of the DSM-IV (Bell, 1994) criteria of major depression on a 4-point Likert scale from 0 (“not at all”) to 3 (“nearly every day”) over the previous 2 weeks. The PHQ-9 is recommended by the DSM-5 working group of the American Psychiatric Association as an instrument to measure the severity of major depression according to the new DSM-5 criteria. The total score ranges from 0 to 27. The PHQ-9 has excellent reliability, as well as criterion, construct, factorial, and procedural validity (Löwe et al., 2004). A score up to 4 indicates the absence of depression, scores of 5–9 represent mild, scores of 10–14 represent moderate and scores of 15 and higher represent severe depression (Löwe et al., 2004). For the German general population, normative data for the PHQ-9 are available for both genders and different age groups (Kocalevent et al., 2013).

#### Generalized Anxiety Disorder Scale

We use the validated German version of the Generalized Anxiety Disorder Scale (GAD-7) (Spitzer et al., 2006; Löwe et al., 2008), the 7-item generalized anxiety module from the PHQ. The GAD-7 is based on the most prominent diagnostic features of the DSM-IV diagnostic criteria for generalized anxiety disorder and has excellent reliability, as well as criterion, construct, factorial, and procedural validity. The 7 items assess frequency of core generalized anxiety disorder symptoms within the past 2 weeks. Items are scored on a 4-point Likert scale ranging from 0 (“not at all”) to 3 (“nearly every day”) with a total score ranging from 0 to 21. A score up to 4 indicates the absence of generalized anxiety disorder, scores of 5–9 represent mild, scores of 10–14 represent moderate and scores of 15 and higher represent severe anxiety symptom levels (Löwe et al., 2008). For the German general population, normative data for the GAD-7 are available for both genders and different age groups (Hinz et al., 2017).

#### Fear of Progression Questionnaire – short form

We use the validated German short form of the Fear of Progression Questionnaire (FoP-Q-SF) (Mehnert et al., 2006a) to measure fear of cancer progression and recurrence in patients and relatives (Zimmermann et al., 2011). The 12-item measure is based on the Fear of Progression Questionnaire (FoP-Q) (Herschbach et al., 2005). The FoP-Q-SF encompasses affective reactions, partnership/family, work, loss of autonomy and coping reactions and has a high internal consistency (α = 0.87). The FoP-Q-SF items are scored on a 5-point Likert Scale ranging from 1 (“never”) to 5 (“very often”). The total FoP-Q-SF score ranges from 12 to 60 and a score of 34 and above indicates a clinical level of FoP (Herschbach et al., 2010). Higher values indicate higher levels of fear of progression.

#### European Organization for Research and Treatment of Cancer Quality of Life Questionnaire

We measure cancer related quality of life (QoL) using the validated German version of the European Organization for Research and Treatment of Cancer Quality of Life Questionnaire Core 30 (EORTC-QLQ-C30) (Aaronson et al., 1993; Schwarz and Hinz, 2001). This instrument is widely used and has excellent reliability and validity. The EORTC-QLQ-C30 contains 30 items, 28 of which are rated on a 4-point Likert scale that ranges from 1 (“not at all”) to 4 (“very much”) and clustered to 5 functioning scales (physical, role, social, cognitive, and emotional functioning); and 3 symptom subscales (fatigue, pain, and nausea and vomiting) and 6 single symptom items (dyspnea, sleeping problems, loss of appetite, constipation, diarrhea, and financial problems). General health and global QoL are rated on 2-items with a 7-point Likert scale that ranges from 1 (“very poor”) to 7 (“excellent”). The raw values are transformed into a range of values from 0 to 100. Higher scores in the functional subscales and global QoL subscale represent better functioning, whereas higher scores in symptom subscales and items reflect a greater extent of symptom distress and worse QoL. For the German general population, normative data for the EORTC-QLQ-C30 are available for both genders and different age groups (Schwarz and Hinz, 2001).

#### Short-Form Health Survey

We use the validated German version of the Short-Form Health Survey (SF-8) to measure general health-related QoL (Ware et al., 1999), a questionnaire which was originally a short-form health survey with 36 questions. The SF-8 comprises eight dimensions of QoL: general health (GH), physical functioning (PF), role physical (RP), bodily pain (BP), vitality (VT), social functioning (SF), mental health (MH), role emotional (RE), and two summary scores for physical (PCS) and mental health (MCS). Items are rated on 5-point and 6-point scales. The raw values are transformed into a range of values from 0 to 100. Higher scores indicate better health-related QoL. For the German general population, normative data for the SF-8 are available for both genders and different age groups (Ellert et al., 2005).

### Partnership aspects and social relationships

#### Illness-specific Social Support Scale – short version

To measure positive support and detrimental social interaction we use the short validated German version (ISSS-8) (Ullrich and Mehnert, 2010) of the Illness-specific Social Support Scale (ISSS) originally developed by Revenson and Schiaffino (1990) and adapted to German language by Ramm and Hasenbring (2003). The validated ISSS-8 captures social interactions perceived as positive and supportive (4 items) or as distressing or harmful (4 items). Items are scored on a 5-point Likert scale ranging from 0 (“never”) to 4 (“always”). Internal consistencies are satisfying to very good (Ullrich and Mehnert, 2010). Higher sum scores in both subscales indicate higher characteristic values.

#### Dyadic coping inventory

We use the validated Dyadic Coping Inventory (DCI) (Bodenmann, 2008; Ledermann et al., 2010), a 37-item self-reporting instrument designed to measure perceived communication and dyadic coping (support each other, cope together and delegate stress to each other in times of overload) that occurs in close relationships when one or both partners are stressed. All Items are rated on a 5-point Likert scale that ranges from 1 (“very rarely”) to 5 (“very often”). The total DCI score is a sum of items 1 through 35. Items 36 and 37 are evaluation items and not included in this study. A DCI total score <111 represents a dyadic coping below average; DCI between 111–145 a dyadic coping in the normal range and a DCI total score >145 a dyadic coping above average. The evaluation of the German translation of the scale provide evidence of internal consistency for the overall scale (α = 0.91) and the individual subscales (German α range 0.61–0.86) (Bodenmann, 2008; Ledermann et al., 2010).

#### Quality of marriage index

We use the validated German version of the Quality of Marriage Index (QMI) (Norton, 1983; Zimmermann et al., 2019), a brief self-report instrument, to measure global perceptions of relationship satisfaction. The scale consists of six positively worded items. Five items are rated on a 7-point Likert scale, ranging from 1 (“strong disagreement”) to 7 (“strong agreement”). The final item, a general estimation of the relationship satisfaction, is rated on a 10-point Likert scale, ranging from 1 (“very unhappy”) to 10 (“very happy”). Total scores range from 6 to 45, with higher scores reflecting better overall marital quality. The cutoff score is 34. The German QMI demonstrates good item characteristics and excellent reliability (α = 0.94) (Zimmermann et al., 2019).

#### Loneliness Scale

To measures perceived feelings of loneliness and social isolation, we use the validated 3-item short version of the Loneliness Scale (LS-3) (Hughes et al., 2004), developed from the revised UCLA Loneliness Scale (Russell et al., 1980). In the German adaptation used in this study, items are scored on a 5-point Likert scale from 0 (“never”) to 4 (“very often”). A higher sum score indicates higher feelings of loneliness.

#### New Sexual Satisfaction Scale – short version

We use the validated German 12 item short version of the New Sexual Satisfaction Scale (NSSS-SD) (Stulhofer et al., 2010; Hoy et al., 2019), a widely used questionnaire for assessing sexual satisfaction. The questionnaire is originally based on two subscales (ego-centered and partner- and sexual activity-centered sexual satisfaction). For the NSSS-SD a one-dimensional factor structure was confirmed with excellent reliability (α = 0.96) (Hoy et al., 2019). This NSSS-SD measures satisfaction with various aspects of one’s own sexual life. The items are scored on a 5-point Likert scale from 1 (“not at all satisfied”) to 5 (“very satisfied”). The scores of all items are summed up. Higher scores indicate higher sexual satisfaction.

#### Sexual quality of life

We use the Sexual Quality of Life (SQoL) questionnaire to measure quality of sexual life. The 18-item questionnaire captures sexual self-esteem, emotional wellbeing and relationship issues in women (SQoL-F) (Symonds et al., 2005) and men (SQoL-M) (Abraham et al., 2008). In the present study, the 18 items are used in a binary (male/female) form. Each item is rated on a 6-point Likert scale (“completely agree” to “completely disagree”) either in descending order 6 to 1 (items 1, 5, 9, 13, and 18) or ascending order 1 to 6 (remaining items). The values of the items are added to a sum score, which is then transferred to a standardized scale from 0 to 100. A higher score indicates higher SQoL (Symonds et al., 2005).

### Supportive care needs and use of psychosocial support services

#### Supportive care needs survey – single items

We use an adapted form of the German version of the Short-form Supportive Care Needs Survey (SCNS-SF34-G) (Sanson-Fisher et al., 2000; Lehmann et al., 2012). The single items cover the five dimensions of the original SCNS-SF34 (psychological support needs, support needs regarding physical problems and coping with daily living, support needs with regard to the health system and information, support needs with regard to patient care and support, and sexuality needs). Items are scored on a 4-point scale from 1 (“no need”) to 4 (“high need”). Higher scores indicate higher supportive care needs.

#### Psychosocial care needs

To assess patients’ perceived supportive care needs, we use single items that had proven suitable in earlier studies (Mehnert and Koch, 2008; Faller et al., 2016). To determine the need for psychosocial support, we ask participants, “Do you have a need for psychosocial support?” Response options were “yes” or “no.” We further ask participants “Would you accept an offer of psychosocial support?” with a yes/no response format to determine the acceptance of psychosocial support offers. In addition, we ask participants to rate the strength of their wish for talking (a) with clinical psychologist and (b) with a closely person: “Do you currently have the wish to talk about the psychological distress because of your cancer?” with the response options 1 (“not at all”), 2 (“somewhat”), 3 (“quite a bit”), 4 (“strongly”), and 5 (“very strongly”).

#### Opportunity to talk to someone

We ask the participants to rate the possibility of talking (a) with a psycho-oncologist and (b) with a close person: “Do you currently have the possibility to talk about the psychological distress because of your cancer?” with the response options 1 (“not at all”), 2 (“somewhat”), 3 (“quite”), 4 (“strongly”), and 5 (“very strongly”).

Using a 5-point response format ranging from 1 (“not at all”) to 5 (“very well”), we ask participants to evaluate their opportunities of talking about their distress to different persons, including family, friends, physicians, nurses, and psycho-oncologist. We ask patients “How well would you be able to talk to the below-mentioned persons about the psychological distress because of your cancer and cancer treatment?” with response options 1 (“not at all”), 2 (“somewhat”), 3 (“partly”), 4 (“well”), and 5 (“very well”), to be rated for each of the individual persons as indicated above.

#### Utilization of psychosocial support services

To assess patients’ utilization of psychosocial support services, we ask participants “Have you previously utilized *psychosocial* support offers because of your cancer?” Six items further specified support: “psychological counseling/psychotherapy,” “social legal counseling,” “pastoral care,” “self-help groups,” “web based-support offers,” and “other offers.” Response options were “yes” or “no.” We further ask participants to rate how helpful they perceived the services to be when they had used them with the response options 1 (“not at all”), 2 (“rarely”), 3 (“somewhat”), 4 (“quite”), and 5 (“strongly”). We further asked utilization of cancer rehabilitation offers. Response options were “yes” or “no.”

We further ask participants if they had been advised by their partner or a close person to seek professional psychosocial support. Response options were “yes” or “no.” We further ask participants if they had received a recommendation from their doctor to seek professional psychosocial support. Response options were “yes” or “no.” We ask participants to specify which professional group was recommended to them (clinical psychology, social worker, pastor, and other). We also ask participants to indicate where they had sought professional psychosocial support (hospital, rehabilitation clinic, outpatient psychotherapy, outpatient cancer counseling center, and other). If participants had not sought professional psychosocial support, they were asked to indicate the reasons why they had not done so.

#### Attitude toward psychosocial support

We ask participants to rate their attitude toward professional psychosocial support offers due to cancer on a numerical rating scale ranging from 0 (“negative”) to 10 (“positive”).

#### Satisfaction with Comprehensive Cancer Care Questionnaire

We use an adapted version of the validated German Satisfaction with Comprehensive Cancer Care (SCCC) Questionnaire (Esser et al., 2021) to measure satisfaction with medical and psychosocial cancer care. In detail, 32 items address human quality of physicians (interest of physicians toward patient and in his/her psychosocial condition), information (psychosocial support services/alternative possibilities of treatment and their consequences) and accessibility to psychosocial services (psychosocial counseling on supportive care services/psycho-oncological support/legal and financial advice/pastoral support/support in searching for a psychotherapist/support in acquiring coping strategies/support to join self-help or therapeutic groups). Response categories differ between item categories including 1 (“very dissatisfied”) to 5 (“very satisfied”); 1 (“very bad”) to 5 (“excellent”); and 1 (“do not agree at all”) to 5 (“completely agree”). Some items contain the additional response option 0 (“not applicable”). Higher values indicate higher satisfaction.

### Health literacy and health behavior

#### Health Literacy Questionnaire

We measure health literacy using the validated German version of the European Health Literacy Survey Questionnaire (HLS-EU-Q16) (Röthlin et al., 2013), the 16-item short version of the 47-item European Health Literacy Survey Questionnaire (HLS-EU Consortium, 2012). The validated questionnaire has a high internal consistency (Cronbach’s alpha = 0.90). All items are rated on a 4-point Likert scale 1 (“very simple” to 4 (“very difficult”). The answer categories of all items are dichotomized. Answers 1 (“very simple”) and 2 (“fairly simple”) are scored with 0. Answers 3 (“quite difficult”) and 4 (“very difficult”) are scored with 1. The scores are summed up. A sum score ≤8 indicates “insufficient,” 9–12 “problematic,” and ≥13 “sufficient” health literacy.

#### Adherence Assessment Questionnaire

We use the validated German version of the Adherence Assessment Questionnaire (AAQ) (Mueller et al., 2018), a self-reporting instrument to assess the extent of non-adherence in chronic indications. The 11-item AAQ has excellent reliability (α = 0.84 to α = 0.97) as well as a good validity. Eight items are formulated as statements that are rated on a 4-point Likert-scale from 1 (“strongly agree”) to 4 (“strongly disagree”). One additional item is stated as a visual analog scale to show the percentage of adherent days in the last 2 weeks. Moreover, two items measuring a patient’s tendency to answer in a socially desirable way were added to the AAQ. The scores of all items are summed up. Higher scores indicate higher inflexibility and less adherence.

#### Questions for the assessment of health behavior

Patients are asked about their drinking and smoking habits, physical exercise, relaxation techniques, regular intake of medication as well as weight and height for calculating the body mass index (BMI). The questions are adapted from the validated German Questionnaire for the Assessment of Health Behavior (FEG) (Dlugosch and Krieger, 1995) and the report of the Federal Health Monitoring in Germany (Schulze and Lampert, 2006). Items are rated on a 4-point Likert scales from 1 (“never”) to 4 (“daily”) or with the response options “yes” or “no.”

#### Stigma Impact Scale

We use the validated German version of the Stigma Impact Scale (SIS) (Fife and Wright, 2000; Eichhorn et al., 2015) to measure perceived experiences of stigmatization. The 24-items measure the effect on patients and their families of negative social attitudes toward the patients’ health or mental health condition. The SIS consists of four subscales: social rejection (9 items), financial insecurity (3 items), internalized shame (5 items), and social isolation (7 items). All items are rated on a 4-point Likert scale that ranges from 0 (“do not agree at all”) to 3 (“fully agree”). Scale means and a total mean score can be calculated. The SIS has a high internal consistency (α = 0.81 to α = 0.89).

### Meaning and purpose in life

#### Schedule for meaning in life evaluation

We use the validated German language Schedule for Meaning in Life Evaluation (SMiLE) questionnaire to measure meaning in life (Fegg et al., 2008). The SMiLE uses an idiographic approach and allows individuals to choose the life areas that they consider to be important for their own meaning in life. Respondents are first asked to indicate from 3 to 7 areas that actually provide meaning to their lives. In a second step (level of importance), the importance of each area is rated on a 5-point scale, ranging from 1 (“somewhat important”) to 5 (“extremely important”). In a third step (level of satisfaction), the respondents rate their current level of satisfaction with each area on a 7-point Likert scale, ranging from −3 (“very unsatisfied”) to +3 (“very satisfied”). Different indices can be formed, which indicate both the mean weighting of the meaning in life areas and the average satisfaction or dissatisfaction with the individual meaning in life areas.

#### The life attitude profile-revised

We use the validated German version of the Life Attitude Profile-Revised (LAP-R) (Reker, 1992; Mehnert et al., 2007), a questionnaire assessing meaning and purpose in life and motivation to find meaning and purpose in life. Here, we use 20 items of the original 48-item questionnaire covering 6 dimensions: purpose (PU), coherence dimension (CO), choice/responsibleness (CR), existential vacuum (EV), and goal seeking (GS). The dimensions of the German validation have satisfactory to high internal consistencies (Cronbach’s alpha between 0.78 and 0.85). All items are rated on a 7-point Likert scale from 1 (“strongly disagree”) to 7 (“strongly agree”). Higher sum scores indicate higher characteristic values in each subscale.

### Standardized SCID-5 interviewer training

All study interviewers are certified psychologists or physicians familiar with the DSM-5 classification and diagnostic criteria. They complete a mandatory standardized SCID-5 training before the beginning of the study interviews. After the SCID-5-CV training, each study interviewer conducts several test interviews with voluntary subjects, one of which was videotaped and evaluated by a certified psychotherapist responsible for quality assurance at the Leipzig university study center. Evaluation criteria included correct implementation of the SCID-5-CV interview questions, comprehension and interactional factors. Each interviewer received detailed feedback about his/her SCID-5-CV training interviews. Interviewers were not allowed to conduct study interviews until they had ensured that they could conduct the SCID-5-CV correctly.

### Study quality assurance, data safety, monitoring, and bias control

The university study center Leipzig is responsible for coordinating data collection and ensuring the accuracy of the assessment, data collection, and data input. The study center Leipzig also plays a key role of facilitating communication between collaborating study centers using routine monitoring, meetings, and feedback to ensure that tasks are accomplished and problems are addressed.

Each collaborating center receives regular reports showing how well the site is meeting recruitment goals, in terms of total number of participants recruited and study targets set. All incoming data is entered at the study center Leipzig into the study database (SPSS Vs. 27) in a timely manner. It is verified that the recruited participants meet all eligibility criteria. With regard to missing data, data editing, and entry procedures, we generate routine data quality reports to assess missing data and inconsistencies. A study research assistant follows up with patients and relatives who have missing data at baseline or follow-up. Adverse events such as death of a patient are documented.

We aim to estimate possible biases in our sample. Patients who meet the inclusion criteria but decline study participation will be asked to provide information about their sociodemographic and medical characteristics, as well as the reason for declining the study on a voluntary basis. Due to data protection regulations and limited access to patients, data for non-participants are only available for the characteristics sex, date of birth, cancer diagnosis, and reason for non-participation. Other characteristics such as SES, stage of disease or Karnofsky index could not be completely collected. Study participants and eligible non-participants will be analyzed in terms of sociodemographic information including sex, age, and medical characteristics including type of diagnosis and cancer stage. Baseline characteristics and follow-up data between those with and those without missing data will also be compared to estimate bias.

## Statistical methods

### Power calculation

The sample size was calculated based on previous data of 4-week prevalence rates of mental disorders in different strata of patients with cancer, assuming an overall prevalence in this population of 30% (Mehnert et al., 2014). In the study application, our original case number planning was to recruit a total of 2,000 cancer patients and 1,600 relatives. Due to the COVID-19 pandemic, which almost coincided with the start of study recruitment, oncology care, and access in the collaborating cancer centers was significantly restricted over a long period of time, so that recruitment opportunities for the study were also severely limited. Therefore, we had to adjust the sample size to approximately 1,000 patients and 600 relatives. With reference to the relevant literature by Newcombe (1998) and Fleiss et al. (2003), a minimum sample size of *N* = 300 is required, given the expected prevalence of mental disorders in cancer patients of approximately 30% (Mehnert et al., 2014).

### Sample stratification

In order to obtain a representative sample, the SES of the patients was screened on the basis of educational level, occupational status, and income. Following the distribution in the German general population, we aimed to stratify the sample in a ratio of 20% (high SES), 60% (medium SES), and 20% (low SES) (Lampert et al., 2013). Stratification also had to be adapted to the limited patient access and recruitment during the COVID-19 pandemic.

Due to the longitudinal design of the study, a failure rate over the measurement time points must also be considered. In a methodologically similar study with cancer patients, a failure rate of 9% was reported (Voigt et al., 2017). However, higher failure rates can be assumed for patients with lower SES (Baekeland and Lundwall, 1975), which is why we conservatively expect a higher failure rate of 25% here.

### Statistical analyses

Prevalence estimates for mental comorbidity (number of patients, %, and SE) are calculated. We present frequencies including their 95% confidence intervals for the main outcome variables. Multiple logistic regression models will be used to calculate and test adjusted differences in age, sex, study center, or tumor entity. In all of the analyses, the quota distribution by SES is taken into account in the form of appropriate weighting factors.

We analyze associations between categorical variables using the Chi-squared test, and between continuous variables using the Pearson correlation coefficient, respectively. Between-group differences are examined using the independent *t*-test for continuous dependent variables. To identify independent predictors, logistic regression analyses will be performed.

For identifying the function of relatives and mutual dependencies between partner and patient (dyadic coping) actor-partner interdependence models (APIM) are calculated. The APIM takes into account the interdependence between the persons within a dyad with regard to the outcome variables under investigation.

The possible influence of dropouts on the representativeness of the results at later measurement times is investigated through pattern mixture modeling (Little, 1995; Hedeker and Gibbons, 1997).

## Discussion

In view of the demographic change, the increasing cancer prevalence and advances in biomedicine, concepts for the development of high-quality care for cancer patients in all survivorship phases that go beyond the existing aftercare are urgently needed with the aim of improving survival and the management of the physical and psychological long-term and late effects as well as alleviating the occupational and social consequences.

Issues of cancer survivorship and quality of life have been put on the agenda both internationally and nationally by various professional societies and national committees (Nekhlyudov et al., 2019). The American Society of Clinical Oncology (ASCO) published criteria for high-quality cancer survivorship care as early as 2013 (McCabe et al., 2013). In Germany, both the National Decade Against Cancer (NDK), which was proclaimed in 2019 by the Federal Ministry of Education and Research (BMBF) together with the Federal Ministry of Health (BMG) (Bundesministerium für Bildung und Forschung, 2022), and the European Cancer Plan (European Commission, 2021) pursue clear objectives with regard to improving the quality of cancer care and the quality of life of those affected in all survivorship phases.

Psychosocial aspects play an important role in initiatives to improve cancer care; not only from the perspective of patients and those affected, but also from the perspective of health care professionals. Although psycho-oncological interventions significantly reduce psychosocial distress such as anxiety and depression and improve quality of life (Faller et al., 2013; Mulick et al., 2018), many patients face barriers to accessing psychosocial support offers (Faller et al., 2016; Weis et al., 2018). A major barrier to low-threshold access to psycho-oncological support offers are socioeconomic inequalities, but these are not well researched in the cancer population. At the same time, the development and implementation of target group-specific psychosocial support services without rigorous longitudinal empirical data will be a challenge in practice.

The data from our study will provide an opportunity to identify at-risk groups for specific psychosocial needs. While it is likely that the majority of survivors do not need or want intensive psychosocial care, those who are at high risk for psychosocial impairment will require more timely and more intensified care. Data that provide the basis for such a risk-based approach, including the development and implementation of survivorship screening, should help to ensure that survivorship care plans can be tailored to the specific needs of each cancer survivor.

This prospective multi-center observational cohort study has a major focus on increasing quality of care and quality of life in cancer survivors through estimating the prevalence of mental disorders, the frequency and extent of psychosocial distress, perceived needs for psychosocial support and utilization of psychosocial support offers in newly diagnosed cancer patients and their relatives a few weeks after cancer diagnosis and over a follow-up period of 18 months. A strength of the study is the inclusion of relatives, who often face a variety of psychosocial challenges during the cancer diagnosis and survival period (Ernst et al., 2017). They often experience a similar psychosocial burden as the patients (Fujinami et al., 2015). Yet, family members usually receive limited support (Rosenberger et al., 2012). While there are support services for relatives in psycho-oncological care (Kuhnt et al., 2018), these are often limited in time or insufficiently tailored to their needs (Lambert et al., 2012; Sklenarova et al., 2015). In the worst case, this can lead to prolonged stress and maladaptive adjustment within the family system.

Findings on the course of psychological distress and perceived supportive care needs in patients and their relatives in the early survivorship phase, taking into account associated biopsychosocial factors, will promote the target-group-specific development of support offers and cancer survivorship care plans particularly for vulnerable groups.

## Data availability statement

The original contributions presented in this study are included in the article/supplementary material, further inquiries can be directed to the corresponding author.

## Ethics statement

The local ethics committee of all study centers approved the study protocol (AZ: 207/18-ek, SR-EK-536112020, 8533_BO_K_2019, 14/4/21Ü). All patients willing to participate provide written informed consent.

## Author contributions

AM-T, JE, PE, UG, and TZ contributed to the conception and design. All authors contributed to the manuscript writing and approved the submitted version.
