# Erratum: Prevalence of mental disorders, psychosocial distress, and perceived need for psychosocial support in cancer patients and their relatives stratified by biopsychosocial factors: rationale, study design, and methods of a prospective multi-center observational cohort study (LUPE study)

**DOI:** 10.3389/fpsyg.2024.1425365

**Published:** 2024-05-09

**Authors:** Frontiers Production Office

**Affiliations:** Frontiers Media SA, Lausanne, Switzerland

**Keywords:** psychosocial distress, cancer patient, relatives, psychosocial support, psycho-oncology, socioeconomic status, survivorship, mental disorders

Due to an editorial error, the article was erroneously published as an “Original Research” article. The correct article type for this article is “Study Protocol.”

The publisher apologizes for this mistake. The original article has been updated.

